# Community-based participatory research: a lifeline to achieve people-centered care

**DOI:** 10.3389/fpubh.2025.1693459

**Published:** 2025-12-15

**Authors:** William E. Rosa, Jeffersson Santos, Anita E. Agbeko, Crystal L. Barksdale, Scott Carvajal, Denise Dillard, Ronit Elk, Gail E. Emrick, Francesca Gany, Shena Gazaway, Jennifer Leng, Melissa Mazor, Martha Moore-Monroy, Naveen Salins, Kendra Godwin, Nirmala Bhoo-Pathy, Juan P. Borda, Loreto Fernández-González, Erica Mann, Gilla K. Shapiro, Matthew J. Allsop, Eliseo J. Pérez-Stable

**Affiliations:** 1 Department of Psychiatry and Behavioral Sciences, Memorial Sloan Kettering Cancer Center, New York, NY, United States; 2 Edson College of Nursing and Health Innovation, Arizona State University, Phoenix, AZ, United States; 3 Center for Community Health and Engaged Research, Northern Arizona University, Flagstaff, AZ, United States; 4 Grupo de Estudos em Metodologias Assistenciais de Enfermagem, School of Nursing, Universidade Federal de Sergipe, Aracaju, Sergipe, Brazil; 5 Department of Surgery, School of Medical Sciences, Kwame Nkrumah University of Science and Technology, Kumasi, Ghana; 6 Division of Community Health and Population Science, National Institute on Minority Health and Health Disparities, Bethesda, MD, United States; 7 Center for Participatory Prevention, Evaluation and Action Research, Department of Health Promotion Sciences, Mel & Enid Zuckerman College of Public Health, University of Arizona, Tucson, AZ, United States; 8 Southcentral Foundation, Anchorage, AK, United States; 9 Institute for Research and Education to Advance Community Health, Elson S. Floyd College of Medicine, Washington State University, Everett, WA, United States; 10 Center for Palliative and Supportive Care, University of Alabama at Birmingham, Birmingham, AL, United States; 11 Division of Geriatrics, School of Medicine, University of Alabama at Birmingham, Birmingham, AL, United States; 12 Southeast Arizona Health Education Center, Nogales, AZ, United States; 13 Immigrant Health and Cancer Disparities Service, Department of Psychiatry and Behavioral Sciences, Memorial Sloan Kettering Cancer Center, New York, NY, United States; 14 Department of Healthcare Policy and Research, Weill Cornell Medical College, New York, NY, United States; 15 School of Nursing, University of Alabama at Birmingham, Birmingham, AL, United States; 16 Division of Internal Medicine, Icahn School of Medicine at Mount Sinai, New York, NY, United States; 17 Department of Palliative Medicine and Supportive Care, Kasturba Medical College Manipal, Manipal Academy of Higher Education, Manipal, Karnataka, India; 18 Medical Library, Memorial Sloan Kettering Cancer Center, New York, NY, United States; 19 Centre for Epidemiology and Evidence-Based Practice, Department of Social and Preventive Medicine, Faculty of Medicine, Universiti Malaya, Kuala Lumpur, Malaysia; 20 Department of Psychiatry, Temerty Faculty of Medicine, University of Toronto, Toronto, ON, Canada; 21 Servicio de Psiquiatría, Fundación Valle del Lili, Cali, Colombia; 22 Centro Integral del Cáncer, Clinica Las Condes, Santiago, Chile; 23 Global Cancer Disparities Initiative, Memorial Sloan Kettering Cancer Center, New York, NY, United States; 24 African Research Group for Oncology, Ile-Ife, Nigeria; 25 Department of Supportive Care, Princess Margaret Cancer Centre, Toronto, ON, Canada; 26 School of Medicine, University of Leeds, Leeds, United Kingdom; 27 National Institute on Minority Health and Health Disparities, Bethesda, MD, United States

**Keywords:** community-based participatory research, people-centered care, universal health coverage, social justice, health equity, community engagement, participatory research, global health

## Abstract

People-centered care (PCC) represents a key paradigm shift in achieving universal health coverage and closing global public health divides. Amid growing global health disparities, shifts in epidemiological disease burden, and evolving sociopolitical contexts that affect healthcare delivery and research initiatives, there is an urgent need for public health scientists to develop community-rooted research strategies that uphold health promotion principles and sustain PCC. Community-based participatory research (CBPR) is a social justice approach that offers a distinct, equity-driven perspective on operationalizing PCC. CBPR fosters long-term, trust-based partnerships; centers the lived experiences and leadership of underserved populations; and co-develops sustainable health interventions that are culturally attuned to communities. Among participatory and community-engaged approaches, CBPR most closely aligns with—and can directly strengthen—the implementation of PCC principles. This paper presents an interprofessional and internationally relevant analysis of how CBPR can support PCC across clinical, public health, and policy domains. We begin by outlining foundational processes for establishing equitable academic–community partnerships. We then detail exemplar CBPR initiatives with racially and ethnically minoritized populations, as well as rural border and migrant communities, highlighting how these collaborations have advanced PCC goals. These exemplars, structured around key CBPR processes and mapped to PCC principles, form the basis of a conceptual blueprint for action. Next, we present a framework for applying CBPR to promote uptake of the World Health Organization’s integrated model for PCC, emphasizing its relevance with consideration to shifting policy and funding landscapes. Finally, we offer actionable recommendations for clinicians, researchers, community partners, health systems, and policy actors to integrate CBPR across the research continuum. To fully realize PCC in a rapidly changing world, researchers must shift from producing knowledge *about* communities to co-producing knowledge *with* them, ensuring that science is conducted in equal partnership with those most affected by structural health inequities.

## 1 Introduction

Growing global public health disparities, shifts in epidemiological disease burden (e.g., toward noncommunicable and chronic diseases), and evolving sociopolitical contexts that affect healthcare delivery and research initiatives underscore the crucial priority of advancing people-centered care (PCC). PCC emphasizes the health of individuals, families, and communities by placing them at the center of healthcare systems and policies (1–3). This aproach ensures care is tailored to people’s specific needs and preferences more holistically across the lifespan while promoting their active and sustained engagement in the development and delivery of healthcare services (1, 4–6). Rather than considering patients as the sole beneficiaries of care services in hierarchical biomedical models, PCC centers the lived experiences of people to shape health systems and policies (1–3). Yet, echoing the Inverse Care Law—where access to high-quality care falls as need rises—PCC remains scarcest for those who need it most (108, 109). This maldistribution arises due to intersecting structural, social, and economic barriers. For example, in many low- and middle-income countries (LMICs), health systems are underfunded and organized around vertical disease programs rather than integrated PCC models – a key barrier to realizing equitable primary care delivery (7). In rural and remote areas, communities often face limited access to services, workforce shortages, and weak engagement channels (8). Socially excluded groups—such as refugees and migrants—encounter legal insecurity, language and cultural barriers, and systemic discrimination, which erode trust and participation in healthcare (9). These factors combine to perpetuate inequitable access to PCC (10).

Given the range of constraints on accessing PCC for marginalized groups, research seeking to support its advancement requires approaches informed by a social justice perspective; to reduce power imbalances, amplify the voices of historically and perpetually oppressed communities, and decolonize research practices (11–15). In such a model, research serves as a health equity tool that is “for and with people” (16). A range of social justice perspectives offer ethical and analytical frameworks with which to explore and address inequalities in health, wellbeing, and broader social systems [e.g., Distributive Justice (17), Procedural Justice (18), Recognition Justice (19), Structural Justice (20) and Transformative Justice (21)]. Procedural justice offers a powerful ethical and methodological lens through which to embed fairness, transparency, and voice within healthcare research and system design. Community-based participatory research (CBPR) exemplifies this approach by positioning patients, carers, and communities as co-producers of knowledge, rather than passive recipients of care. By addressing epistemic and structural injustices, CBPR enables the co-design of interventions that are more contextually relevant, acceptable, and equitable (22, 23). Embedding CBPR in the development of PCC is, therefore, both a practical and moral imperative to ensure care models are grounded in what matters most to those they intend to serve.

CBPR can bolster co-learning and capacity building among all research participants, conduct studies that align with a given community’s priorities, and ensure that findings will measurably and sustainably contribute to community health improvements (22, 23). Moreover, CBPR promotes equity among all scientific and lay actors, including community members and leadership, researchers, clinicians, and policymakers (22). Aligning with antiracist and decolonizing practices, CPBR empowers communities, particularly those historically neglected and currently marginalized, to iteratively participate in all research phases, from conceptualization to the dissemination of findings, and leverage community assets to strengthen what is possible through the development and broad implementation of community-sourced, evidence-based interventions and policies (24). Through collaborative efforts, CBPR allows partner communities to have agency in defining roles and agendas, shaping the research process, and co-creating knowledge based on their context and experiential wisdom (22, 25). The 11 principles of CBPR (26–28) embody health equity paradigms (24, 29–37). Specifically, CBPR:

- Recognizes community as a unit of identity.
- Builds on strengths and resources in the community.
- Facilitates a collaborative, equitable partnership in all phases of research, involving an empowering and power sharing process that attends to social inequalities.
- Fosters co-learning and capacity building among all partners.
- Integrates and achieves a balance between knowledge generation and intervention for the mutual benefit of all partners.
- Focuses on the local relevance of public health problems and ecological perspectives that attend to the multiple determinants of health.
- Involves systems development using a cyclical and iterative process.
- Disseminates results to all partners and involves them in the wider dissemination of results.
- Involves a long-term process and commitment to sustainability.
- Openly addresses issues of race, ethnicity, sexism, racism, and social class and embodies “cultural humility” (38).
- Works to ensure research rigor and validity but also seeks to “broaden the bandwidth of validity” with respect to research relevance.

We contend that CBPR is a scientific approach ideally positioned to realize PCC while also increasing representation of minoritized groups in clinical trials and global public health, and centering the role of community-academic partnerships in empirical endeavors (39–43). In fact, CBPR has emerged as a commonly cited form of community-engaged research (CEnR) and is arguably the gold standard in demonstrating commensurate and equitable partnerships between researchers and communities (44, 45). Consistent, transparent, and respectful people-centered engagement is the cornerstone of CBPR, crucial for identifying community-identified care needs and priorities (46, 47).

This paper unites community organization leadership, scientists, clinicians, community advisory board members, and policymakers from nine countries and aims to provide an interprofessional and internationally relevant document that rigorously explores CBPR utilization in PCC research and practice. First, we outline processes for establishing and enhancing trusting partnerships with community members, patient advocacy and grassroots organizations, and advisory boards to support CBPR initiatives. Next, we summarize exemplars of collaborative research endeavors with racially and ethnically minoritized populations, as well as rural border and migrant communities that illustrate CBPR co-creation, conduct, and sustainment. These exemplars provide a rare but essential in-depth description of CPBR’s core elements and demonstrate how CBPR practices have directly advanced PCC goals in diverse settings. Third, we offer a blueprint for how CBPR can assist with the uptake of the World Health Organization (WHO) integrated framework on PCC. Finally, we articulate recommendations for a range of actors to integrate CBPR approaches throughout the research continuum and in broader healthcare endeavors. In sum, we suggest that for CBPR to help realize PCC, researchers must shift the collective mindset toward producing science in full partnership with—and not just about—diverse communities in the global world.

## 2 Trust with community organizations and community advisory boards

**Figure 1:**
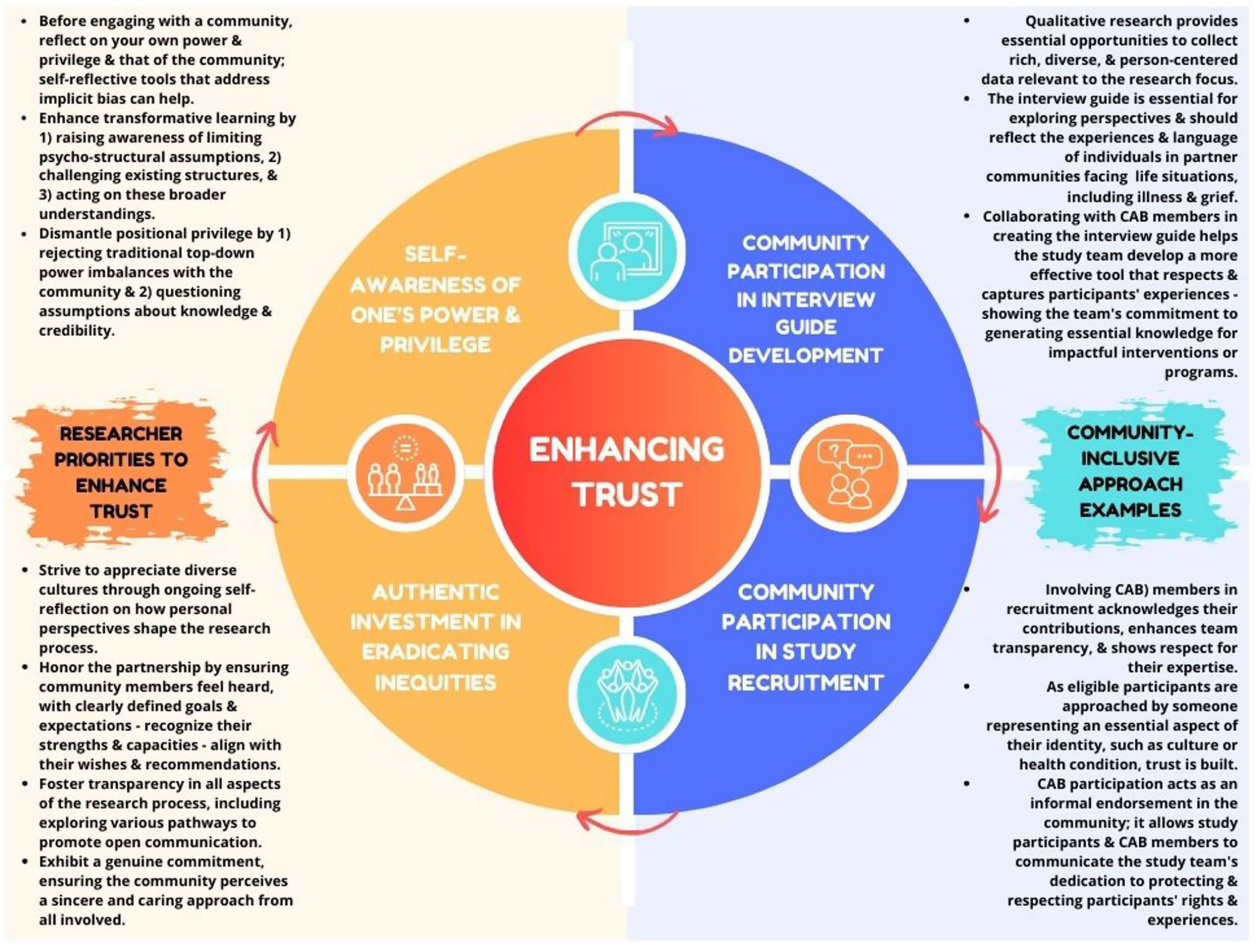
Researcher priorities and community-inclusive approaches to enhance trust.

Building and maintaining trust is the foundation for sustaining long-standing CBPR partnerships (48). A recent scoping review highlighted that trust is linked to mutual respect, including how partners interact by valuing and acknowledging each other’s skills, expertise, and knowledge, as well as their participation in this partnership. Building trust and respect led to increased openness and transparency, enhanced relationships, and created safe spaces for community members and researchers to share personal vulnerabilities. Additionally, several outcome indicators included development of pride and ownership in joint work, transfer of knowledge from the partnership to the community members, clear benefits of the research to the community, increased power sharing in the partnership, mutuality of the benefits of collaboration, and the continued willingness of the community to conduct CBPR (48). Equally important is leveraging opportunities to increase community representation, cultural acknowledgment, and respect (49, 50). Figure 1 provides descriptions of researcher priorities to enhance trust (51–53), as well as examples of community-inclusive research approaches in interview guide development and study recruitment (54, 55).

The need for both enhancing and repairing trust through cross-cultural representation and inclusion is particularly important for both researcher and community partners and especially when working with minoritized, disenfranchised, and historically neglected populations (56). For example, trust building with tribal health systems was essential for the successful piloting of two interventions (i.e., depression management decision support tool and a trauma screening, brief intervention, and referral to treatment process) with Native Nations communities. Steering committees comprised of American Indian and Alaska Native (AI/AN) patients, providers, and leaders guided both efforts. Providing key considerations and recommendations in interviews and focus groups to ensure interventions were feasible, effective, and culturally appropriate (57, 58). Interventions were piloted with AI/AN participants (59, 60) and results shared with patients, providers, leaders, the Native Nations involved, and the broader AI/AN community (61, 62).

Strengthening the diversity and CBPR competencies of research teams is also essential, providing opportunities for researchers to acquire community-centered skills and knowledge, and for all partners to examine how institutionalized, personally mediated, and internalized forms of oppression affect collaborative work. The U.S. National Institute on Drug Abuse funded Culturally-Centered Addictions Research Training (C-CART) program, for instance, trains clinicians and doctoral students in research skills grounded in culturally-centered practices for substance use and substance use disorder prevention and treatment (63). C-CART actively includes scholars from underrepresented groups in the U.S., such as Native Americans, Latinos, African Americans, and LGBTQ+ people, as well as international scholars from Brazil, India, Ghana, and Puerto Rico, among others (63). Building on this training, these scholars are now involved in global research initiatives that honor cultural traditions and actively engage people and their communities in addressing their own health priorities (64–69).

## 3 Community-based participatory research exemplars

Here, we provide in-depth descriptions of CBPR initiatives in diverse communities and settings to describe how these can be operationalized in practice and meaningfully advance PCC goals across various settings. This is key to understanding how these approaches can be adapted and applied to relevant contexts. We present two in-depth exemplars that include a partnership between a comprehensive cancer center and an immigrant population, as well as a multi-decade initiative in the U.S.-Mexico border region. Additional exemplars in rural Ghana and the U.S. rural deep south describe culturally-centered intervention development in the palliative care context for diverse communities.

### 3.1 Memorial Sloan Kettering Cancer Center’s Immigrant Health and Cancer Disparities Center

Almost 25% of the New York City (NYC) population has limited English proficiency (70). Communication is the cornerstone of effective and trusted engagement, care delivery, and research participation. For Memorial Sloan Kettering Cancer Center (MSK) Immigrant Health and Cancer Disparities Center’s (IHCD’s) work, a CBPR approach has been central to advancing people-centered care by directly addressing linguistic (i.e., bridging varied NYC language communities), cultural, and structural barriers to equity in cancer care. IHCD has developed evidence-based linguistic engagement platform methodologies for research inclusion, including multilingual measurement tools, a process for transcreation of study materials into participants’ languages, and multilingual data collection methods (i.e., focus groups, individual and group interviews, surveys). The IHCD team includes fluent speakers of Spanish, Mandarin, Cantonese, Russian, Ukrainian, Bengali, Arabic, French, and Korean, among other languages. In addition, IHCD researchers have developed and launched a platform for remote, simultaneous medical interpreting, also known as United Nations (UN)-style interpreting. These innovations emerged through sustained CBPR partnerships and reflect a commitment to language justice and inclusive research.

Deep, long-standing partnerships with immigrant and underserved communities underpin the IHCD’s model. IHCD engages the communities where they work, meet, play, pray, eat, and network (e.g., WhatsApp), through (a) community-embedded institutions, e.g., community-, occupation-, faith-based organizations, and consulates, (b) community providers and safety net systems, (c) local colleges, and (d) patient organizations. These partnerships utilize learning collaboratives to bidirectionally build capacity and facilitate the exchange of learning, data, skills, and ideas. IHCD CBPR activities often germinate through these learning collaboratives, through our Community Advisory Board (CAB) meetings, through our needs assessment processes, and/or as a result of community-informed and engaged service delivery projects. Project-specific CABs: (a) help to define research questions and/or service delivery priorities and share in decision-making around study design and implementation, (b) develop protocols to engage the community to participate in research and service, (c) provide context for results interpretation, (d) disseminate-return research results to the community, and (e) participate in the selection of resultant clinical and policy priorities. The CBPR processes are informed by a Steering Committee that is co-led by the community, comprising clinical, population sciences, and basic and translational researchers, as well as providers, with institutional and community champions, and shared leadership and decision-making. Activities are coupled with program and policy partners to facilitate the translation of research findings into practice and policy.

To enhance research participation, IHCD developed the Integrated Cancer Care Access Network (ICCAN), a comprehensive, community-engaged, people-centered resource network and patient navigation program for underrepresented cancer patient populations. ICCAN integrates MSK sites with a network of over 360 community- and faith-based organizations, other cancer clinics, healthcare providers, cancer support organizations, social services organizations, and policy makers. ICCAN can help address system barriers, social risks, and essential needs of low-income, predominantly immigrant and minoritized, community members and patients who often have limited English proficiency (LEP). It operates in 15 cancer clinics and multiple community organizations within MSK’s catchment area. ICCAN facilitates cancer care access; ameliorates the financial toxicity of cancer treatment; addresses patient needs such as health insurance, safe housing, stable income, access to healthy foods, legal services, and psychosocial support; bridges the linguistic and digital divide; provides education and training for, and brings the community’s voice to, researchers, clinicians, and care teams, and is another vital platform for sharing research findings with the community. This integrative approach exemplifies how CBPR can institutionalize PCC by aligning services with community-defined needs and preferences.

The Taxi Network further illustrates how these activities coalesce. Half of the taxi drivers and many of the app-based for-hire vehicle (FHV) drivers in the U.S. work in the MSK catchment area. Taxi and FHV driving is a stressful, sedentary occupation, with poor access to healthful food, and shared cardiovascular disease and cancer risk factors, and poor health outcomes. Over 91% of the NYC taxi/for-hire vehicle driver population are immigrants, mainly from South Asian, francophone West African, and Latin American countries (71, 72). The West African community is at disproportionately greater risk for prostate cancer, and for colorectal cancer that is especially aggressive (73, 74). Drivers of all origins are at higher CRC risk due to poor diet and sedentary lifestyle (75–77). Working with partner organizations, IHCD and the South Asian Council for Social Services facilitated the establishment of the CBPR Taxi Network over ten years ago. Several research priorities have been iteratively identified over the past decade, including cardiovascular health, diet, and physical activity, financial strain leading to health strain, stress and mental health, and cancer screening. These have led to NIH-funded randomized controlled trials and optimization studies on physical activity, health care access, hypertension, and diet and physical activity. The study results have led to numerous program and policy changes.

IHCD’s CBPR approach has not only improved research inclusivity but also embedded PCC principles—trust, relevance, responsiveness, and co-leadership—within cancer care systems. These exemplars demonstrate how CBPR can operationalize PCC in diverse, urban, multilingual, and historically underserved populations.

### 3.2 The US-Mexico border region: University of Arizona and community health workers

For over 35 years, the University of Arizona has been active in CBPR in the US-Mexico border region. This long-standing partnership has demonstrated how CBPR can serve as a powerful vehicle for advancing PCC, particularly through the elevation and institutionalization of programs for community health workers (CHWs) programs. This work includes tracing back to a pilot study showing the promise of CHWs to improve birth outcomes in Mexican-origin farmworkers (78) that laid the groundwork for what is now an evidence-based global health model for reducing maternal and child health inequities. Subsequent iterations have embedded CHWs across every county in this diverse state (79), illustrating how community-anchored roles can sustain equitable care delivery across time and geography.

Throughout this research, community partners, and specifically CHWs, have been at the core. This includes in problem identification, project conception and implementation, and shared dissemination efforts to impact research, policy, and practice. Methods have included qualitative approaches to give voice to historically neglected populations and uncover unanticipated (to researchers) themes (80–82), and in some cases, informed quantitative measurement (83, 84). Quantitative methods, particularly useful in applied settings, including conjoint analysis and propensity score matching, have been employed to address research questions originating from community partners, leading to funded collaborative projects (85, 86). The use of existing health systems and public health surveillance data—without adding burdens to participants—has enabled the team to evaluate structural and policy-level impacts over extended time periods (79, 87). These methods align with PCC principles by minimizing extractive research practices while yielding actionable evidence on system-level disparities, improving chronic disease self-management, and informing the expansion and sustainability of the CHW workforce.

As important as a focus on disparities—and there are critical health and social inequities in Mexican-origin border populations in the U.S.— is an orientation to health advantages and community strengths and assets. Despite socioeconomic challenges and systemic discrimination, communities demonstrated ability to cope with adversity, resilience, strong social ties, and health practices that respect their community traditions—dimensions essential to PCC but often overlooked by dominant health systems (84, 86). Recently, ethnographically informed interviews have illustrated that community members are attuned to the connection between stress and health in ways not typically conceived or measured by dominant health systems or prior research, and they have articulated health adaptations. This work may inform interventions aimed at reinforcing and sustaining protective social and cultural resources within these communities. Still, it may also highlight strategies for promoting population health in other communities across the U.S., Mexico, or perhaps across continents. For example, recent longitudinal work in Germany has identified parallel health patterns in their immigrant communities, as observed in North America (88). There may be bidirectional and transferable knowledge and approaches that are useful across nations and continents.

The emphasis on community capacity building represents one of the most transformative outcomes of CBPR. Two long-time partners in Arizona-Sonora border health—grassroots organizations where CHWs highly, or completely, shape their strategic goals and daily activities, are part of the backbone of a new and novel federal initiative. They are among the 25 community organizations supported nationwide in the inaugural rollout of Community Partnerships to Advance Science for Society (ComPASS) program. With ten years of expected efforts, ComPASS affirms that community-rooted organizations—particularly those with CBPR experience—are uniquely positioned to organize around structural determinants of health and strengthen existing community resources to promote enduring improvements to people’s well-being (89).

One current effort, Proyecto Juntos (Project Together), is a CBPR initiative to address structural barriers to mental health care and stigma in rural and border counties in Southeast Arizona. A community-based organization long active in CHW workforce development and local, binational, health and social action, is the leader and prime awardee. Uniquely, a community-based organization—not an academic or healthcare institution—serves as the prime awardee and lead investigator, reflecting CBPR’s commitment to redistributing power and centering community leadership.

Of note, two arts-based qualitative methods are being implemented to complement other qualitative and quantitative research strategies in the initial project phase. The creation and sharing of Digital Stories (90) has been effective in setting the tone for sensitive discussions with equal voice among all partners. Grounded in the concept that rivers hold symbolic significance across many cultures, River of Life (91) is an activity where partners draw, map, and converse about their own journey to and with this coalition. Along with promoting cohesion and understanding, the products--which start with butcher paper and color markers—have illuminated upstream barriers such as stigma in the community and with providers, workforce challenges that may be met with more CHWs and enhancing mental health training in multiple languages, the need for culturally relevant resources, and addressing transportation challenges. They have also identified areas where community resources on both sides of the border can be better leveraged, as well as where new partners from non-traditional health sectors can increase the coalition’s impact and reach in reducing health inequities. While highly ambitious, it is also humbling; there is a strong belief that great community benefit will result from reducing mental health stigma at multiple levels, expanding services available, and being more responsive to community needs and preferences. We hope in 10 years to show through common commitment and strategic activities like that described to expand the collation and keep it engaged, connected and moving forward, the current effort may have the same type of sustained, positive and institutionalized improvement around mental health like we observed for CHWs programs on improving maternal and child health and chronic disease outcomes that we began to see over two decades ago in this region.

### 3.3 Culturally-concordant palliative care in Ghana and the U.S. rural deep south

Culturally-based intervention development is central to CBPR and ensuring the provision of PCC. Palliative care – as a model of person- and family-centered care – requires cultural sensivity, respect, and inclusion throughout the serious illness continuum, including at end of life. Table 1 describes culturally-centered intervention development using CBPR in rural Ghana and among racially diverse communities in the U.S. deep south living with serious illness.

**Table 1:** Culturally-centered community-based participatory research initiatives for and with marginalized and underserved communities with serious illness.

Exemplar (A) culturally-based care preferences for patients with life-limiting illness in rural Ghana	Exemplar (B) culturally-concordant palliative care intervention among African American and white patients in the U.S. deep south
Ghana’s collectivist culture centers family and community cultural values (94), an essential concept to understand to provide goal-concordant care for patients with life-limiting illness. Komfo Anoyke Teaching Hospital (KATH) is a tertiary hospital located in Kumasi, a city of 1.5 m people in Ghana. To elicit the cultural values, preferences, and goals of care of patients’ family caregivers who had received end-of-life care at KATH through focus groups, CBPR principles were used to develop an 8-member CAB who advised on focus group implementation (e.g., meeting site, discussion topics, ethical considerations, recruitment methods) (95). Emergent themes from the focus group included health system problems: unreliable physician access, high out-of-pocket care costs, timely diagnosis, unmanaged pain and symptoms, and poor physician-patient-caregiver communication. Three cultural values were identified: caregivers’ pivotal role in caring for loved ones; discussion of prognosis requiring involvement of community members, and key role of God (i.e., faith in illness and dying processes). This pilot study provided insights into values, preferences, and goals of care, and provided community caregivers the opportunity to participate in exploring issues that directly affected them.	Culturally-centered care is essential in palliative care to ensure minority patient’s needs are met (97, 105, 106). Yet, palliative and end-of-life care has been historically rooted in white, middle class cultural and religious values that emphasize individualism (96) and is often unable to deliver values-aligned care for African Americans with serious illness (e.g., family-centered decision making) (97, 98). In the first study to implement a culturally based palliative care intervention (99), culturally-based values and care and communication preferences for treating providers were determined for separate white and African American groups using CBPR principles and equitable CAB partnerships. Findings between the two groups either (a) diverged: e.g., African Americans believed that hope and miracles were always a possibility regardless of medical test results with God as the decider and expressed a strong distrust of healthcare systems and physicians—experiences white community members did not share; (b) were similar but varied: e.g., although religion and church were important in both groups, African American community members considered the church as unreservedly central to every aspect of life. Culturally based recommendations were then incorporated into two distinct consult protocols for palliative care providers (100) to follow in the study (e.g., in the African American protocol, the palliative care provider would acknowledge God as the ultimate decider, adding affirming language about hope and the possibility of miracles).

## 4 CBPR to drive people-centered care

This section describes how CBPR can support the implementation of the WHO’s integrated framework for people-centered care (1). The WHO framework on integrated PCC provides four key policy and action domains for driving and sustaining the ideological shift within health systems toward PCC: (1) individuals, families, and communities; (2) health practitioners; (3) health care organizations; and (4) health systems. Although strengthening health systems will allow strategies at the health consumer, health practitioner, and organizational levels to be increasingly effective and sustainable, there must be continuous collaboration between these four PCC domains and proactive engagement of systems’ leadership. To ensure CBPR fulfills its potential in advancing PCC, actors across the research continuum must embed participatory values and methods at every stage of design, implementation, and dissemination. Our team generated a consolidated blueprint to guide this integration, linking CBPR processes with the WHO’s PCC action domains (Table 2). Key cross-cutting recommendations include: (1) Institutionalize community leadership structures, such as co-chaired steering committees and funded CABs, from the outset of research partnerships; (2) Prioritize flexible and iterative research designs that allow for community-defined outcomes and timelines; (3) Align funder and institutional metrics with indicators of partnership quality, mutual benefit, and long-term sustainability; (4) Embed training on cultural humility, procedural justice, and CBPR methods across clinical, public health, and implementation science curricula; and (5) Translate participatory processes into policy advocacy and service reform, particularly in settings serving historically marginalized populations.

**Table 2:** Community-Based Participatory Research Blueprint to Realize the Key Messages of the World Health Organization Integrated People-Centered Care Framework.

**WHO Integrated PCC Framework Actors**	**Key messages**	**How CPBR helps realize each message**
**Individuals, families, and communities**	Improving health literacy Enabling communication and negotiation skills Improving self-efficacy and ability to self-care Capacity building of the voluntary sector, community-based organizations and professional organizations Developing social infrastructure that supports community participation Developing community leaders who advocate and support community involvement in health service delivery	A1. Allows researchers to gain insight into the existing knowledge and skills of individuals, families, and communities regarding health topics, including cultural practices and informal health networks. A2. Aids in identifying gaps in health literacy within communities that may not be apparent to researchers but can be revealed through community engagement. A3. Facilitates the co-creation of research questions that align with the community’s health literacy needs, the development of interventions tailored to the community's culture and utilizing its resources to improve health literacy, and the dissemination of health information that is easily understood by community members. B1. Facilitates transparent communication among community members and various actors, including researchers, providers, and policymakers, to facilitate the creation of research that addresses the community's specific needs. B2. Enables community members to engage in constructive conversations with providers and policymakers, empowering them to play an active role in determining the best approaches to enhancing their health. C1. Conducting research that addresses community health needs, collaborating on interventions utilizing community resources, and enabling communities to control their health. C2. Collaborative creation of strategies and tools designed to promote self-care, such as health education programs and health support groups. D1. Fostering collaboration between community health partners, including academic institutions, healthcare facilities, non-governmental organizations, and advocacy groups, to co-develop research questions that align with community priorities. D2. Strengthening relationships between community partners to promote shared goals and providing them with resources such as relevant health data, tailored health promotion interventions, and self-care tools to enhance their capacity to support community health through healthcare delivery and advocacy. E1. Establishment of a precedent for community participation in decision-making processes regarding their health E2. Setting a stage for developing empowered communities capable of engaging in dialogues with policymakers concerning community health priorities E3. Ensure that the most effective practices are integrated into health services and policy planning and implementation. F1. Empower community members to assume leadership positions in research, fostering the development of essential skills such as communication with providers and policymakers, negotiation, advocacy, and public speaking. F2. Enable community leaders to effectively advocate for and promote community engagement in policy and health service delivery.
**Health practitioners**	Develop capacity for holistic and compassionate care Enhance commitment to quality, safe and ethical services	A1. Foster an environment encouraging individuals, families, and communities to articulate their expectations for comprehensive and empathetic care from healthcare providers. A2. Develop culturally sensitive approaches that providers must incorporate into healthcare delivery, effective communication techniques for building solid relationships with individuals, and avenues for provider participation in community activities. A3. Facilitate the development of training initiatives and other plans to enhance providers' capabilities for delivering comprehensive and empathetic care. B1. Community engagement at every stage of health service development and evaluation ensures that healthcare provision is aligned with community needs and priorities, ultimately improving quality and safety. B2. Promote ethical healthcare by ensuring that interventions have community consent and are culturally-centered.
**Health care organization**	Create a conducive and comfortable environment for people receiving health care and for health practitioners Ensure effective, efficient, ethical, safe, coordinated and quality care through establishing and strengthening multidisciplinary care teams Introduce and strengthen models of care Enhance leadership capacity of health services managers in championing people-centered health care	A1. Allow identification of structural and environmental features that are considered important by community members for creating a welcoming atmosphere within healthcare facilities. A2. Engage with the community to uncover facility layout, signage, or accessibility that directly shapes people’s healthcare experience. A3. Gather input from healthcare providers, enabling them to identify structural features and workflow processes that they believe can promote a more efficient, comfortable, and supportive care environment. B1. Co-design of participatory studies to comprehend the barriers and facilitators for effective multidisciplinary collaboration and coordination of care within healthcare facilities. B2. Development of initiatives and interventions aimed at promoting comprehensive healthcare. C1. Support the implementation of innovative healthcare models tailored to meet communities' specific needs. C2. Integrate community feedback into service evaluation to foster ongoing quality improvement, enhancing the effectiveness of existing healthcare models. C3. Align with local health priorities, making them more viable and adaptable over time. D1. Fostering strong connections between health services managers and the communities they serve for gaining a deeper understanding of local health needs. D2. Motivating managers to lead initiatives that prioritize people-centered care approaches and empower them to advocate for policies and resources that support people-centered care.
**Health Systems**	Developing and strengthening primary care and the primary care workforce Building a stronger evidence base on ways to improve health care and the health system itself to achieve better health outcomes Enhancing the oversight of professional standards, implementing public accountability measures for health services organizations, delivery and financing, and monitoring and addressing patient and community concerns about healthcare quality. Assist individuals who have encountered adverse events and uphold the confidentiality of patient information.	A1. Developing a strategy for the co-design, implementation, evaluation, and reform of primary care based on the preferences and priorities of those who are meant to benefit from it. A2. Developing policies that steer more comprehensive and robust primary care services with community participation as a core feature. A3. Community involvement in shaping training programs for current healthcare professionals to ensure comprehensive healthcare delivery can influence the training of future healthcare professionals in higher education institutions. B1. Contribute significant insights to the existing literature on communities' health needs and assets that can inform the development, restructuring, execution, and assessment of health policies and customized healthcare delivery protocols to improve overall population health. B2. Implementing financial incentives to encourage positive provider behaviour and enhance access and financial security for the entire population. C1. Ensure that communities can freely express their preferences for healthcare delivery, particularly in their interactions with healthcare providers. C2. Help shape policies that establish standards for primary care delivery to these communities, assist in developing protocols to assess providers' proficiency in caring for them, and ensure rational use of technology. D1. Explore the experiences of individuals who have encountered adverse events within the healthcare system to gather valuable insights into the risk and protective factors associated with these events. D2. Research to inform the creation of customized protocols within healthcare systems to prevent adverse incidents or reduce their impact.

## 5 Recommendations

For CBPR to thrive and realize its potential to advance PCC, science in traditional academic, lay, and multi-sector settings should be open to reconceptualization and restructuring. Such efforts have been underway for a long time in national research strategies and investments aimed at understanding the fundamental drivers of health disparities and developing strategies to reduce them (92). For instance, The National Institute on Minority Health and Health Disparities (NIMHD), National Institutes of Health (NIH) created the Community-Based Participatory Research (CBPR) Program in 2005 to address the need for improved transdisciplinary intervention research methods and approaches that address health disparities, and to strengthen the science of community engagement in addressing disparities among socially disadvantaged populations. Starting with initiatives that provided 11 years of phased funding for the implementation of interventions to reduce disparities using CBPR principles, NIMHD has been intentional in developing funding opportunities that support meaningful academic-community partnerships and recognize community assets. Over the course of the CBPR Program, over 130 awards have been made to research institutions.

As CEnR methodologies have become more common in NIMHD-funded projects, the need for an initiative focused solely on promoting the CBPR approach has decreased, but it remains crucial. Recommendations generated from the NIMHD Science Visioning process completed in 2019 indicated the need to shift from individual-level and researcher-derived interventions to more community-derived, structural, multi-level, and multi-sectoral interventions to improve minority health sustainably and reduce health disparities. NIMHD broadened its focus to support community-engaged research approaches that utilize the NIMHD Research Framework to assess and intervene on health determinants beyond the individual level through community-level intervention funding opportunities (93). The COVID-19 pandemic response and the current NIH Common Fund initiative, which provides direct funding to community organizations, further evidence the commitments to and successes with this approach.

To develop active and engaged CBPR partnerships, community members must be valued as scientists in their own right. One such initiative is the Tisch Cancer Institute’s Community Scientist Program (107), launched in 2022, a network of community partners and patient advocates that are integrated throughout the research continuum. Community scientists are lay advocates who have experienced cancer themselves or been a cancer care partner and trained in research through the Community Scientist Institute, along with additional training depending on their area of interest (e.g., research methods). Their research roles range from developing research ideas to dissemination, partnering on proposal development, internal grant review, manuscript and abstract writing, focus groups, and interpreting and disseminating findings. The Community Scientist program enhances equity-focused and community-responsive research, workforce diversity, and community-engaged curriculum for trainees and fellows, contributing to and fostering a meaningful learning community for those interested in cancer research. This program demonstrates how Community Scientists play a pivotal role in promoting people-centered care by acting as bidirectional bridges between researchers and communities.

**Figure 2:**
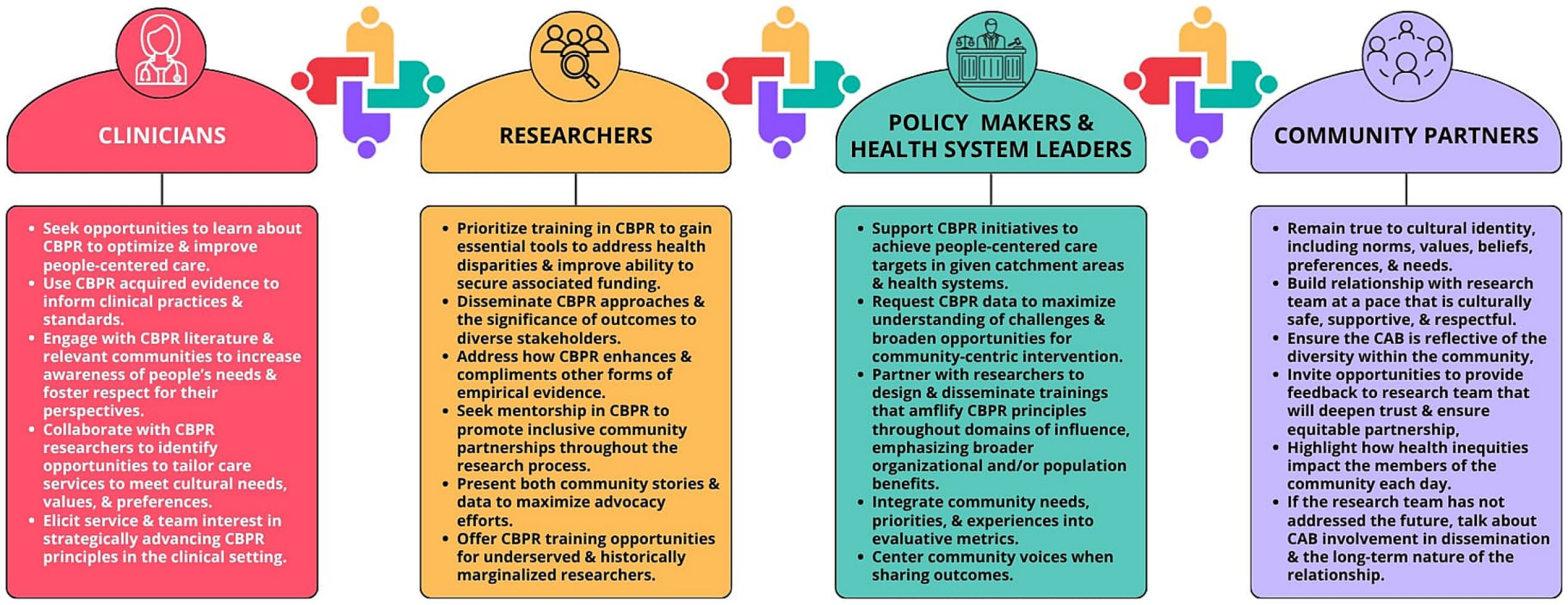
Additional recommendations for diverse actors to promote community-based participatory research.

**Figure 3:**
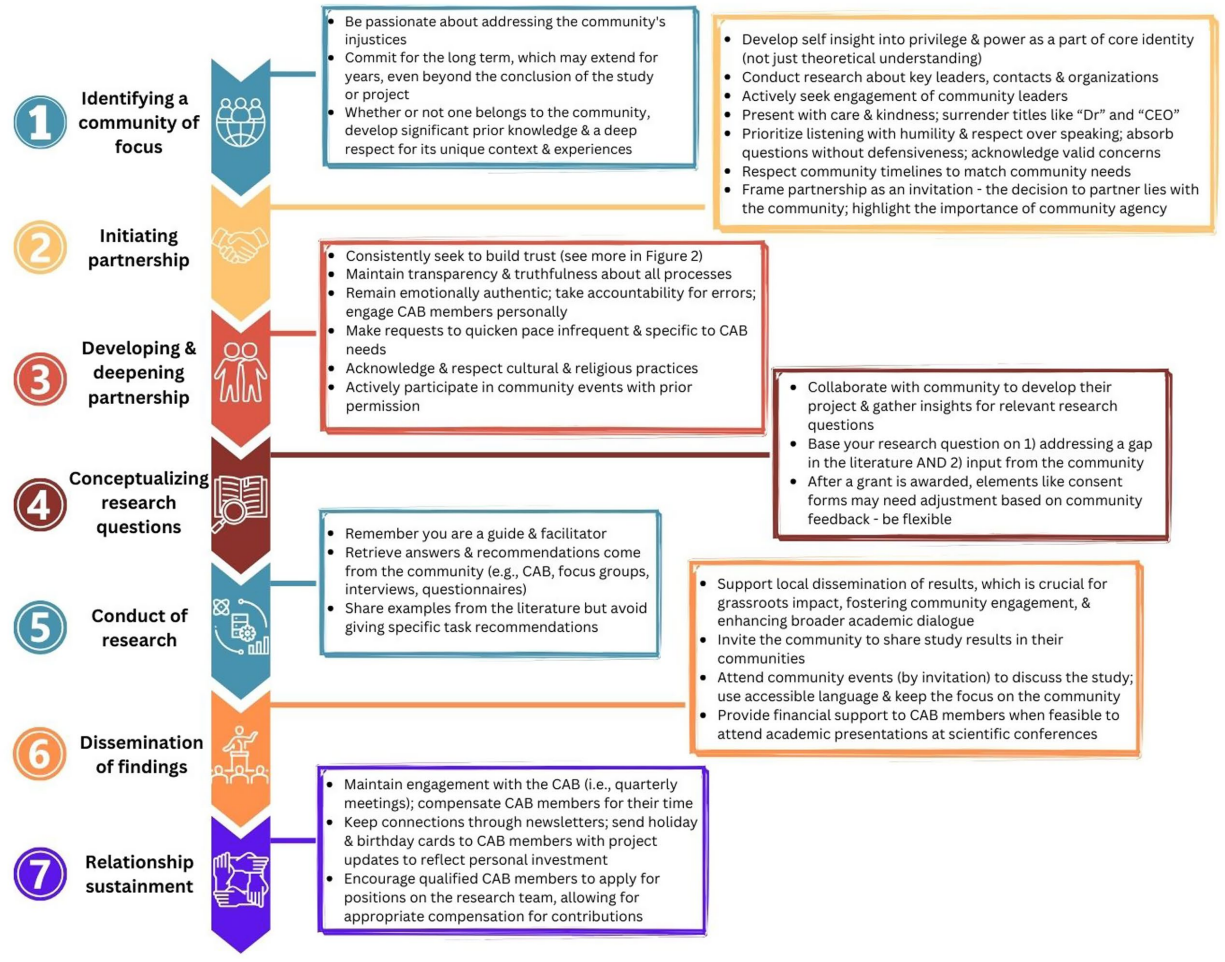
Recommendations for investigators to incorporate participatory values and methods across the research continuum.

Figures 2, 3 offer further detailed recommendations for researchers and multisectoral stakeholders for embedding participatory values and methods across the research continuum.

## 6 Sustaining CBPR amid evolving sociopolitical contexts

Shifts in funding structures for health initiatives and research have challenged the continuation of CBPR programs. In this context, sustaining CBPR requires strategic adaptation and diversification of funding and structural models. One possible strategy is partnering with community-based organizations as primary awardees, positioning them as institutional leaders rather than subcontractors. This model, exemplified by NIH’s ComPASS initiative, redistributes power and aligns leadership with community priorities (101). Another approach may involve exploring alternative funding sources, such as philanthropic and institutional sources. For example, the Robert Wood Johnson Foundation supports participatory, health equity-focused research through programs such as Evidence for Action and Research to Advance Racial and Indigenous Health Equity, which mandate community-centered leadership and governance. The W. K. Kellogg Foundation similarly funds community-led initiatives aimed at advancing racial equity and health. University seed grants, health system social innovation funds, and public health partnerships may also provide early-stage resources to initiate CBPR (102). Small-scale investments—like mini-grants for CABs or jointly co-designed pilot projects—may catalyze trust and shared ownership, making CBPR resilient even amid challenging sociopolitical contexts.

## 7 Synergies and tensions between CBPR and PCC

CBPR offers concrete ways to embed community members in shared governance structures and decision-making processes. Nonetheless, significant tensions exist between CBPR and mainstream PCC implementation. System integration remains a key challenge: while PCC is often deployed within existing clinical frameworks, CBPR may critique or resist these very systems when they perpetuate inequity. Temporal alignment is another point of tension. Health systems favor scalable, time-efficient interventions, whereas CBPR requires sustained relationship-building and iterative engagement, often over years (103). Power dynamics also diverge: PCC initiatives may still operate through top-down institutional structures, whereas CBPR insists on horizontal, community-led governance, which some systems may resist. Finally, evaluation poses a challenge: PCC often relies on standardized metrics and patient-reported outcome measures, whereas CBPR prioritizes community-defined indicators of success, which may not align with conventional evidence hierarchies (104). In politically constrained environments, these tensions are heightened. As federal CBPR programs are scaled back or canceled, it becomes even more important to support CBPR through local partnerships, flexible funding mechanisms, and community-centered governance. While initial investments for CBPR may be modest—often just enough to support early relationship-building and shared agenda setting—sustained support is needed to embed these partnerships within systems of care and research. Exemplars from this manuscript demonstrate that once relationships and shared infrastructure are established, CBPR can persist and evolve across policy cycles, reinforcing PCC even in the face of retrenchment.

## 8 Conclusion

CBPR is the “gold standard” (44, 45) for co-creating equitable partnerships with communities, ensuring that research and its translation to practice and policy are people-centered and, thus, “for and with people” (16, 24). Longstanding CBPR partnerships highlighted in this paper exemplify the core tenets of people-centered care: trust, shared leadership, mutual capacity building, and responsiveness to locally defined needs and priorities. By aligning research processes with community values within broader clinical, public health, and policy systems, CBPR strengthens both the practice and institutionalization of PCC. As interest in CBPR grows—both as a rigorous scientific approach and a vehicle for advancing health equity—researchers, policymakers, funders, and community actors must work together to ensure that CBPR is equitably resourced, appropriately implemented, and meaningfully sustained across diverse settings. Especially in times of political and structural uncertainty, CBPR offers a durable framework for protecting and advancing people-centered approaches to care.
